# A tribute to Carsten Schmuck

**DOI:** 10.3762/bjoc.17.190

**Published:** 2021-11-29

**Authors:** Jochen Niemeyer, Ivo Piantanida, Thomas Schrader

**Affiliations:** 1 Faculty of Chemistry (Organic Chemistry) and Centre for Nanointegration Duisburg-Essen (CENIDE), University of Duisburg-Essen, Universitätsstraße 7, 45141 Essen, Germany; 2 Division of Organic Chemistry & Biochemistry, Ruđer Bošković Institute, PO Box 180, 10002 Zagreb, Croatia

Carsten Schmuck (Figure 1) was Professor of Organic Chemistry at the University of Duisburg-Essen (UDE), where we had the pleasure to work with him during the past years. Carsten unexpectedly passed away in 2019, leaving a huge gap for his family, friends, colleagues, students and many more. His unexpected departure left us deeply moved and we soon realized that Carsten not only had a profound impact on our lives, but certainly also on the lives of many other people. While the scientific contributions in this special issue are meant to honor Carsten´s scientific legacy, we would like to use this editorial to take a more personal view and remember his influence on all the people he interacted with – including the three of us.

Jochen Niemeyer, Ivo Piantanida, Thomas Schrader

**Figure 1:**
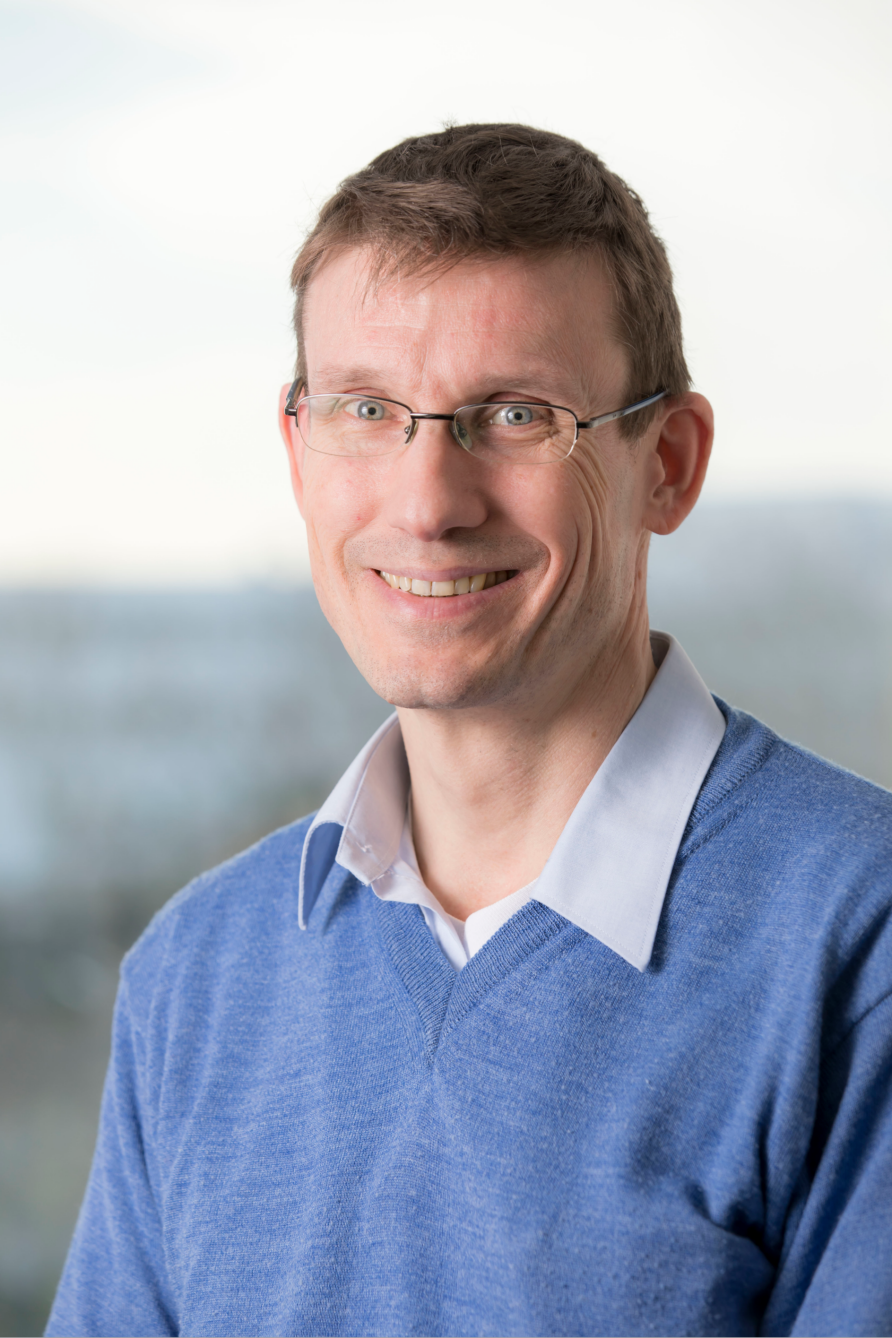
Carsten Schmuck.

## A dedicated teacher and mentor

For Carsten, teaching was never a burden in his busy schedule, but something he profoundly enjoyed. He passed his knowledge and – more importantly – his passion for chemistry to students and mentees of different ages, be it high-school students, university students, Ph.D. students, postdocs or assistant professors working under his supervision.

His love for chemistry started early: Carsten himself was a participant in the Chemistry Olympiad when he was a high school student and remained to be an active member in this community. Not only did he serve as a referee during the Olympiads, but he also organized seminars to prepare the participants (and he did not hesitate to go to the supermarket on his own to buy snacks and drinks for this event).

For his university students, Carsten always took his job description as “Hochschullehrer” (literally: “university teacher”) seriously. He enjoyed his classroom teaching all the way from basic chemistry to advanced supramolecular chemistry, and he always had an open ear for the questions and concerns of students, both in his role as a professor and as the Dean of Chemistry. But he did not stop there: When he had the feeling that the current chemistry textbooks did not fully meet the needs of his students, he simply wrote a new textbook. His books on “Chemistry for Medical Students” and “Basic Organic Chemistry” are outstanding examples of Carsten’s ability to explain chemical concepts in an understandable fashion, without oversimplifying the science behind it. In Essen and beyond, these textbooks have become the gold standard for undergraduate teaching, so everyone is well-advised to read “The Schmuck” when diving into the world of (Organic) Chemistry for the first time. On top of all that, he also took on the task to write an updated version of the classical organic chemistry encyclopedia “Beyer/Walter”, together with his long-time colleagues Tanja Schirmeister and Peter Wich. This outstanding piece of work was rightfully honored with the Literature Prize of the Chemical Industry Fund in 2017.

Carsten’s enthusiasm for science also spread to his team. Over his career, he mentored more than 40 Ph.D. students, more than 10 postdocs and numerous research students in his group. His style of leadership was always full of trust: he gave his co-workers the independence to develop and carry out challenging research projects and saw himself as a companion, not so much as a supervisor, throughout the project. While this was not always easy for his co-workers when projects turned out to be difficult, Carsten definitely reached his aim of fostering the next generation of researchers with an independent and open-minded way of thinking. The fact that most (if not all) of his former co-workers have successfully transitioned to fulfilling jobs in academia, industry or other fields was very important to Carsten, and he made it a tradition to invite all former and current groups members to a summer barbecue at his home every year.

Last but not least, Carsten hosted a number of assistant professors in his group, and I had the pleasure to be one of these (together with Michael Giese and Jens Voskuhl). On top of his support in terms of lab space and finances, Carsten was a great mentor who always managed to find the right balance between kind words of support and words of criticism when necessary (and when asked for). He never failed to write a message of support before important presentations (“Good luck at the Chemiedozententagung!”) and always conveyed his clear belief that we were on a good track in our scientific careers. It turns out Carsten was right, we only wished that he would have been here to see this happen. We will continue to honor Carsten by returning some of his support to the next generation of scientists, especially to the last generation of Ph.D. students that are currently graduating from the Schmuck group.

Jochen Niemeyer

## A scientific globetrotter

Carsten Schmuck had very intensive international collaborations with many group leaders all over the world, easily visible from scientific publications and from a recent review article [1]. Moreover, Carsten also took a prominent part in several formal EU collaboration schemes, giving a notable contribution to the development of the noncovalent and supramolecular recognition research in the European Research Area (ERA). His personal and scientific engagement was equally intensive with excellent, well-established scientists, and new groups from small scientific communities; for instance, starting collaboration in the early 2000s with our group from the Croatian Research Institute Ruđer Bošković.

My personal experience working with Carsten very likely reflects the experience of other colleagues and co-workers all over the world. Our first meeting and scientific discussion took place in 2004 within a COST D31 action, at which Carsten was the head of the work group WG0018/05, entitled “Guanidinium as an important anchor group in supramolecular recognition”. At that time, the focus of WG0018/05 was mostly on nonaqueous anion recognition systems and constructs, but during our several discussions and mutual visits, we agreed to try Carsten’s guanidiniocarbonylpyrrole (GCP) cation system in our group for DNA/RNA recognition. In that way, we started our collaboration and exchange of students which was, thanks to Carsten’s great personality, very pleasant and friendly. Moreover, due to Carsten’s profound knowledge in noncovalent recognition systems, it also turned out to be very efficient. We continued our collaboration through the next COST Action CM1005, including other research groups, and thus broadening the research interest, followed by Carsten being a partner in the largest Croatian FP7 project, REGPOT-2012-2013-1 (http://www.innomol.eu/), as well as in several joined bilateral projects, funded by Germany and/or Croatia.

A typical course of these 15 years of our collaboration (for sure also familiar for all other colleagues collaborating with Carsten) was marked by a very intensive interplay in exchange of scientific ideas and knowledge. Carsten continuously gave a lot of attention and constructive questions about fine details in targeted binding sites of biomacromolecules (various types of DNA and RNA, or proteins), as well as already known small molecules binding to these targets. Then, taking into account particular features of “Carsten’s GCP” unit, or related analogues, he would propose several feasible structural modifications, which could provide novel properties of scientific interest. Finally, upon mutually agreeing on the next generation of new compounds, he would always take the agreement most seriously, dedicate a researcher from his group to the task, support his/her visits to Croatia and also visits of our researchers to his lab, thus encouraging the exchange of experience. Upon completion of studies, Carsten would very efficiently take part in summarizing and writing up the results, thus keeping pace with ongoing studies, Ph.D. or postdoc student needs and obligations, as well as project deadlines. Such very intensive collaboration resulted in 16 jointly published papers – for both groups the most intensive and productive international collaboration. I am sure that a similar pattern took place with all other of Carsten’s collaborative projects and research, marking him an internationally well-recognized scientist and expert in supramolecular chemistry and beyond.

Ivo Piantanida

## A great colleague

My first encounter with Carsten was at his poster on a conference: We were both working on our habilitation and soon realized: “Wow, we really share the same interest (recognition of ionic species with designed receptor molecules)!” Immediately, we decided to exchange ideas, with the consequence that our groups visited each other, at that time between Würzburg and Marburg. In 2008, Carsten was called on his chair at the UDE, and from the beginning we had a very good atmosphere among all colleagues from the Organic Chemistry division, who occasionally met for the “docent tea” at Carsten’s office. He quickly made friends with other professors in chemistry and started collaborations even in fields such as empirical educational research. But his social competence went beyond that: Each year at Nikolaus (Dec 6), Carsten filled stockings with sweets for each of his co-workers; he initiated a common Christmas party for the entire Organic Chemistry division and on faculty retreats he cooked for all professors on our faculty excursions, even in the confined space of the kitchen on a traditional sailing ship!

Carsten always had a view for the larger perspective. Soon he initiated talks with several institutions, which led to the establishment of three Junior Professor positions in Organic Chemistry at the UDE. It was a privilege in 2010 to start the nucleus of our later Collaborative Research Center 1093 with him, which we called “Supramolecular Chemistry on Proteins” and which he directed as Vice Speaker, with great enthusiasm. In 2011, Carsten organized the first “SupraChem” conference, the beginning of an ever increasing national platform on supramolecular chemistry, attracting young scientists and advancing the influence and reputation of this relatively young discipline throughout Germany.

Finally, it was undeniably a strength of Carsten to engage in academic self-governance and university politics, in the best sense of the word. He therefore volunteered twice to be the Dean of the Chemistry faculty, with an eye for justice and prosperity of the entire faculty. On the scientific level, Carsten was elected member of the “Fachkollegium” of the DFG, the most influential body with respect to deciding about grant applications and criteria for their evaluation. I am sure that all my colleagues will agree that Carsten deserves our highest respect for his dedication and his consideration of all different interests and perspectives. It was a privilege to have him as a close colleague – and friend.

Thomas Schrader
